# Supplementary material for: The Comparative Efficacy of Treatments for Children and Young Adults with Internet Addiction/Internet Gaming Disorder: An Updated Meta-Analysis
Source: Int J Environ Res Public Health. 2022 Feb 24;19(5):2612. doi: 10.3390/ijerph19052612 (PMC8909504; doi:10.3390/ijerph19052612)
Supplement: Supplementary file 1 [file ijerph-19-02612-s001.zip › ijerph-1565799-supplementary.pdf]

Table S1

Papers included in the updated meta-analysis

No	Author, (Year)	Journal title
1	Cao FL et al, (2007)	Control study of group psychotherapy on middle school students with Internet overuse
2	Li G & Dai XY, (2009)	Control study of cognitive–behavior therapy in adolescents with internet addiction disorder.
3	Shao Z et al, (2015)	The effects of fluoxetine in treating middle school students with depressant internet addiction disorder
4	Wei QX, (2008)	The role of psychological nursing intervention in the treatment of internet addiction
5	Yang FR et al, (2005)	The effect of integrated psychosocial intervention on 52 adolescents with internet addiction disorder
6	Liao XC, (2010)	Comprehensive intervention on internet addiction of middle school students: an analysis of 142 cases
7	Wu LZ et al, (2007)	Treatment on 27 adolescents with internet addiction by 2/100 Hz Han's acupoint nerve stimulator
8	Pan SJ et al, (2010)	Efficiency of Electroencep Halographic biofeedback treatment in middle school students with internet addiction disorder
9	Liao YR et al, (2012)	The effects of Adlerian group counseling on adolescents with internet addiction
10	Li & Dai, (2009)	Control study of cognitive–behavior therapy in adolescents with internet addiction disorder
11	Bai & Fan, (2007)	The effects of group counseling on internet-dependent college students
12	Du YS et al, (2010)	Longer term effect of randomized, controlled group cognitive behavioural therapy for internet addiction in adolescent students in Shanghai
13	Han DH et al, (2009)	The effect of methylphenidate on internet video game play in children with attention-deficit/hyperactivity disorder
14	Yang R et al, (2005)	Comprehensive intervention on internet addiction of middle school students
15	Zhu TM et al, (2009)	Clinical effect of electro-acupuncture combined with psychologic interference on patient with internet

		addiction disorder
16	Yeun YR et al, (2016)	Effects of psychosocial interventions for school-aged children's internet addiction, self-control and self-esteem: meta-analysis
17	Liu J et al, (2017)	Effects of group counseling programs, cognitive behavioral therapy, and sports intervention on internet addiction in east Asia: systematic review and meta-analysis
18	Winkler et al, (2013)	Treatment of internet addiction: a meta-analysis
19	Liu QX et al, (2015)	Multi-family group therapy for adolescent Internet addiction: exploring the underlying mechanisms
20	Yang Y et al, (2017)	Electro acupuncture treatment for internet addiction: Evidence of normalization of impulse control disorder in adolescents
21	Han DH et al, (2012)	Bupropion in the treatment of problematic online game play in patients with major depressive disorder
22	Park JH et al, (2016)	Effectiveness of atomoxetine and methylphenidate for problematic online gaming in adolescents with attention deficit hyperactivity disorder
23	Mun SY et al, (2015)	Effects of an integrated internet addiction prevention program on elementary students' self-regulation and internet addiction
24	Lien T.-C., (2007)	A study of the effectiveness in applying solution-focused group counseling to Internet-addicted adolescents
25	Huang Z et al, (2010)	Effects of interpersonal group counseling on college students with computer gaming addiction
26	Khazaei et al, (2017)	Positive psychology interventions for internet addiction treatment
27	Hui Li et al, (2017)	Effect of electro-acupuncture combined with psychological intervention on mental symptoms and P50 of auditory evoked potential in patients with internet addiction disorder
28	Kim SM et al, (2012)	Combined cognitive behavioral therapy and bupropion for the treatment of problematic on-line game play in adolescents with major depressive disorder

29	Han DH et al,(2012)	The effect of family therapy on the changes in the severity of on-line game play and brain activity in adolescents with on-line game addiction
